# Suicide in adolescents exposed to the youth justice system: A 22-year retrospective data linkage study from Queensland, Australia

**DOI:** 10.1192/j.eurpsy.2023.706

**Published:** 2023-07-19

**Authors:** R. Borschmann

**Affiliations:** Centre for Mental Health, University Of Melbourne, Carlton, Australia

## Abstract

**Introduction:**

Little is known about the epidemiology of suicide in young people exposed to the youth justice system (YJS).

**Objectives:**

We aimed to estimate the suicide rate in a large cohort of young people exposed to the YJS in Australia, and to identify the demographic/criminogenic risk factors associated with these deaths.

**Methods:**

Data relating to all young people who had any contact with the YJS in Queensland between January 1993 and December 2014 (N=49,228) were linked to Australia’s National Death Index. We calculated the incidence rate of suicide within the cohort, stratified by sex and Indigenous status. Poisson regression was used to assess the change in suicide rates over time. Crude mortality rates (CMRs) were calculated for all-suicide and method-specific suicides, both overall and within subgroups.

**Results:**

Of the 48,228 participants, 1452 (3%) died during the follow-up period. For 31% (458) of decedents, the cause of death was suicide. The proportion of deaths due to suicide was highest for Indigenous females (37.9% of all deaths), followed by Indigenous males (36.8%), non-Indigenous males (30.1%) and non-Indigenous females (25.8%). Hanging was the most common method of suicide (83%).

**Conclusions:**

The disproportionately high incidence of suicide following contact with the YJS is a cause for concern. There is a pressing need to better understand the trajectories of young people after discharge from the YJS. This missing epidemiological knowledge would inform targeted, preventive interventions to be implemented during the window of opportunity when these vulnerable young people are under the care of the YJS.

**Disclosure of Interest:**

None Declared

